# Identification of RUNX1T1 as a potential epigenetic modifier in small‐cell lung cancer

**DOI:** 10.1002/1878-0261.12829

**Published:** 2020-11-27

**Authors:** Tian He, Gary Wildey, Karen McColl, Alyssa Savadelis, Kyle Spainhower, Cassidy McColl, Adam Kresak, Aik Choon Tan, Michael Yang, Ata Abbas, Afshin Dowlati

**Affiliations:** ^1^ Department of Biochemistry School of Medicine Case Western Reserve University Cleveland OH USA; ^2^ Division of Hematology and Oncology Case Western Reserve University Cleveland OH USA; ^3^ Department of Pathology University Hospitals Cleveland Medical Center Cleveland OH USA; ^4^ Department of Biostatistics and Bioinformatics Moffitt Cancer Center Tampa FL USA; ^5^ University Hospitals Seidman Cancer Center Cleveland OH USA

**Keywords:** E2F pathway, epigenetics, exome sequencing, RUNX1T1, small‐cell lung cancer

## Abstract

Small‐cell lung cancer (SCLC) can be subgrouped into common ‘pure’ and rare ‘combined’ SCLC (c‐SCLC). c‐SCLC features a mixed tumor histology of both SCLC and non–small‐cell lung cancer (NSCLC). We performed targeted exome sequencing on 90 patients with SCLC, including two with c‐SCLC, and discovered *RUNX1T1* amplification specific to small cell tumors of both patients with c‐SCLC, but in only 2 of 88 ‘pure’ SCLC patients. *RUNX1T1* was first identified in the fusion transcript AML1/ETO, which occurs in 12%‐15% of acute myelogenous leukemia (AML). We further show higher expression of *RUNX1T1* in the SCLC component of another c‐SCLC tumor by *in situ* hybridization. *RUNX1T1* expression was enriched in SCLC compared with all other cancers, including NSCLC, in both cell lines and tumor specimens, as shown by mRNA level and western blotting. Transcriptomic analysis of hallmark genes decreased by stable *RUNX1T1* overexpression revealed a significant change in E2F targets. Validation experiments in multiple lung cancer cell lines showed that *RUNX1T1* overexpression consistently decreased *CDKN1A* (p21) expression and increased E2F transcriptional activity, which is commonly altered in SCLC. Chromatin immunoprecipitation (ChIP) in these overexpressing cells demonstrated that RUNX1T1 interacts with the *CDKN1A* (p21) promoter region, which displayed parallel reductions in histone 3 acetylation. Furthermore, reduced p21 expression could be dramatically restored by HDAC inhibition using Trichostatin A. Reanalysis of ChIP‐seq data in Kasumi‐1 AML cells showed that knockdown of the RUNX1T1 fusion protein was associated with increased global acetylation, including the CDKN1A (p21) promoter. Thus, our study identifies *RUNX1T1* as a biomarker and potential epigenetic regulator of SCLC.

## Graphical abstract

*RUNX1T1* is highly expressed in ‘combined’ and ‘pure’ SCLC via genetic amplification or greater transcription, respectively. RUNX1T1 interacts with the *CDKN1A* (p21) promoter and is associated with reduced histone H3 acetylation, resulting in decreased p21 expression and increased E2F transcriptional activity in lung cancer cell lines. Thus, RUNX1T1 is both a potential marker and epigenetic modifier in SCLC.

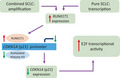

**Abbreviations**
AML: acute myelogenous leukemia
CCLE: Cancer Cell Line Encyclopedia
ChIP: chromatin immunoprecipitation
c‐SCLC: ‘combined’ SCLC
GEMM: genetically engineered mouse model
GSEA: gene set enrichment analysis
HDAC: histone deacetylase
IHC: immunohistochemistry
NE: neuroendocrine
NSCLC: non–small‐cell lung cancer
SCLC: small‐cell lung cancer
TCGA: The Cancer Genome Atlas
TMA: tissue microarrays

## 1 Introduction

Small‐cell lung cancer (SCLC) is a high‐grade neuroendocrine (NE) tumor that represents about 15% of the total lung cancer population. SCLC is very different from non–small‐cell lung cancer (NSCLC), which includes all other forms of lung cancer, in terms of its pathology, molecular features, and therapeutics. SCLC is the most highly metastatic lung cancer, and surgical intervention is rare [1, 2, 3]. Chemotherapy remains the backbone of treatment in SCLC because it is not associated with any recurrent targetable genomic alterations, such as *EGFR* mutation in NSCLC. Despite a robust overall sensitivity toward chemotherapy, patients with SCLC demonstrate high relapse rates, leading to the poorest survival rates among all patients with lung cancer.

There are two clinically recognized subgroups of SCLC, called ‘pure’ and ‘combined’, which are also dissimilar from one another. Combined SCLC (c‐SCLC) is characterized by mixed tumor histology, meaning a combination of SCLC and a subtype of NSCLC, in the same tumor or in two different synchronous tumors. Large cell carcinoma is the most frequent NSCLC associated with c‐SCLC [4]. Importantly, the 2015 World Health Organization (WHO) classification lists c‐SCLC under the category of SCLC, and not NSCLC. This is because c‐SCLC tumors are treated as ‘pure’ SCLC, with platinum doublet therapy, rather than as NSCLC. Despite a reported incidence varying from 2 to 28% in all of SCLC, little is known about c‐SCLC [4, 5, 6]. While a hallmark of ‘pure’ SCLC is *RB1* loss of function, with expected increase in E2F activity, this is not known for c‐SCLC.

*RUNX1T1* (*RUNX1* partner transcriptional co‐repressor 1), also commonly called MTG8 (myeloid transforming gene chromosome 8) or ETO (eight twenty‐one), is a member of the ETO family, which also includes MTG16 and MTG‐R1 [7]. The *RUNX1T1* gene was first identified in the fusion transcript AML1/ETO, generated by a translocation between chromosomes 8 and 21, which occurs in 12%‐15% of acute myelogenous leukemia (AML) [8]. In addition to its well‐known role in AML, RUNX1T1 functions on its own as a transcriptional co‐repressor, mainly by interacting with different histone deacetylases (HDACs) [7, 9]. Biochemical studies have shown that RUNX1T1 can physically bind to several co‐repressors, including Sin3, N‐COR, and SMRT, and participates in different HDAC complexes [9, 10, 11]. Furthermore, RUNX1T1 is reported to be involved in various context‐dependent processes, such as neuronal differentiation of radial glial cells, microglia proliferation, endothelial angiogenesis, adipocyte differentiation, and NOTCH signaling [12, 13, 14, 15, 16].

Here, we report the specific amplification and expression of *RUNX1T1* in patients with SCLC, in particular c‐SCLC. Because the role of RUNX1T1 by itself as a nonfusion protein has rarely been examined in cancer, including lung carcinoma, we sought to determine whether RUNX1T1 could contribute to the SCLC phenotype and if so, its mechanism of action.

## 2 Materials and methods

### 2.1 Patients and genomic profiling

We have an ongoing IRB‐approved institutional database that includes all patients with SCLC diagnosed or treated at our institution. The current retrospective analysis included all patients seen at our institution from 2013–2019 and is an extension of previous studies [17, 18, 19]. We collected data on age, sex, race, smoking status, stage, and genomic alterations (see Table S1). We used the FoundationOne CDx sequencing platform (Cambridge, MA, USA), which currently interrogates the complete exomes of 324 genes, as well as introns of select genes involved in gene fusions/rearrangements, to obtain mutation profiles from formalin‐fixed paraffin‐embedded (FFPE) tumor biopsy specimens [20]. The great majority of specimens were diagnostic and pretreatment. Patients who did not have next‐generation sequencing of tumor DNA were excluded from our study. The site of tumor biopsy was recorded, and all cases received pathological confirmation of disease.

In the two index c‐SCLC patients of the 90 patient cohort, two separate biopsy specimens were submitted for genomic analysis after histologic review by a thoracic pathologist to assign pathology. For PID #508, an initial biopsy was taken from the diaphragm on 1/20, diagnosed as SCLC, and sent out for sequencing. A second biopsy from the lung was taken on 1/29 and diagnosed as large cell and adenocarcinoma, but not sequenced. A third biopsy from a lymph node was taken at disease recurrence on 8/4 after two different treatment regimens, diagnosed as adenocarcinoma and sent out for sequencing. For PID #512, on 2/5 a lung biopsy was taken, diagnosed as poorly differentiated adenocarcinoma, and sent out for sequencing. A second lung biopsy was taken on 2/26, diagnosed as SCLC, and sent out for sequencing. The third c‐SCLC tumor specimen analyzed by *in situ* hybridization was from a resection and graded as 80% SCLC and 20% adenocarcinoma‐acinar type.

Our IRB approval includes genomic and molecular testing of archival human specimens and conforms to the standards set by the Declaration of Helsinki.

### 2.2 Transcriptomic database and analysis

The *RUNX1T1* mRNA expression profile (Affymetrix, Santa Clara, CA, USA) in cell lines was downloaded from the Cancer Cell Line Encyclopedia (CCLE) (https://portals.broadinstitute.org/ccle). The RNA‐Seq data of 31 patients with SCLC obtained in the study by Rudin *et al*., 2012, were downloaded from the European Genome database. RNA‐Seq data were analyzed as previously described [19]. Briefly, RNA‐Seq reads were aligned to the hg19 genome using TopHat. The counting of reads mapped to each gene was performed using HTSeq, and Cufflinks was used to calculate FPKM (fragment per kilobase per million). Transcriptome profile of the other cancers (BRCA: breast cancer, CR: colorectal cancer, GBM: glioblastoma, LUAD: lung adenoma NSCLC, LUSC: lung squamous NSCLC, PRAD: prostate adenoma cancer, SKCM: skin melanoma cancer) was downloaded from the TCGA data portal (https://gdc.cancer.gov/) and analyzed as described above.

### 2.3 Cell lines culture and chemical treatment

Four NSCLC cell lines (H1650, PC9, H1299, and A549) and nine SCLC cell lines (DMS79, H2171, H1694, SHP77, H82, H446, H69, SW1271, and H841) were purchased from ATCC (Manassas, VA, USA) and cultured in DEME/F12 or RPMI‐1640 with 10% FBS and 5% GlutaMAX. Cells were revalidated by STR profiling if used for more than 3 years.

Trichostatin A (TSA) (Cell Signaling Technology, Danvers, MA, USA, #9950) was prepared in DMSO as a stock solution of 4 mm. Cell lines H1299 and SW1271 were seeded in 100 mm plates and treated by TSA at 400 nm and 2 mm, respectively. Cells were harvested after six hours of treatment and lysed for western blotting.

### 2.4 Western blotting

Protein lysates (50 µg) were prepared and analyzed on 4–20% Criterion gels (Bio‐Rad, Hercules, CA, USA), as described previously [21]. Primary antibodies used were RUNX1T1 (Novus Biologicals, Centennial, CO, USA, #NBP2‐55747), p21 Waf1/Cip1 (Cell Signaling Technology, Danvers, MA, USA, #2947), and beta‐actin (Sigma, St. Louis, MO, USA, #A‐5441).

### 2.5 Generation of *RUNX1T1* overexpressing cell lines

To perform *RUNX1T1* overexpression, cells were seeded into 96‐well, 24‐well, or 12‐well plates and then transduced with lentiviral particles for FLAG‐tagged RUNX1T1 (GeneCopoeia, Rockville, MD, USA, catalog #LPP‐T3109‐Lv101‐100) at 5 MOI with polybrene. Twenty‐four hours after transduction, cells were changed to fresh medium and then selected under G418 treatment (1–3 mg ml^−1^) for at least 2 weeks.

### 2.6 *RUNX1T1* CRISPR/Cas9 knockout

*RUNX1T1* guide RNA (AAGAGTTCGCACCCTCGTTC) on a pLentiCRISPRv2 vector was purchased from GenScript (Piscataway, NJ, USA) and delivered into cells using lentiviral transduction, as described above. Cells were selected under puromycin at 0.5µg/ml for about two weeks to generate stable cell lines.

### 2.7 Generation of *RB1* knockdown cell lines

To stably knockdown *RB1*, lentiviral plasmid pLKO.1‐RB1‐shRNA19 was purchased from Addgene (Watertown, MA, USA, catalog # 25640). Cells were seeded in a 6‐well plate and transduced with lentivirus and selected under puromycin at 0.5µg/ml, as described previously [19].

### 2.8 Microarray and pathway analysis

For each cell line, total RNA was isolated using TRIzol (Invitrogen/Thermo Fisher Scientific, Waltham, MA, USA) and RNeasy mini kits (QIAGEN, Germantown, MD, USA), and transcriptome profiles were determined using Clariom S gene microarrays (Thermo Fisher Scientific). Microarray data analysis was performed by the Gene Expression Core facility of the Case Comprehensive Cancer Center. Briefly, data were normalized using the Affymetrix power tools using the robust multiarray average method. Multiple probe sets were collapsed to gene‐level expression data using the GSEA collapse dataset function. Pathway analysis was performed by Gene Set Enrichment Analysis (GSEA) using hallmark gene sets. Heatmaps and hierarchical clustering for genome‐wide expression profiling were generated using Clustvis. Clustering of both cell lines and genes were done using correlation distance and average linkage between groups after rows were centered and unit variance scaling applied.

### 2.9 E2F luciferase reporter assay

The Cignal E2F Reporter Assay Kit from QIAGEN was used. This kit provides a mixture of a firefly luciferase reporter construct containing multiple repeats of a canonical E2F‐binding site and a constitutively driven *Renilla* luciferase plasmid. Cells were seeded into 96‐well plates for overnight incubation and then transfected with 100ng of the reporter mixture per well using Lipofectamine 2000 (Invitrogen/Thermo Fisher Scientific) following manufacturer’s instructions. Forty‐eight hours after transfection, cells were lysed and assayed using a Dual‐Luciferase Reporter Assay system (Promega, Madison, WI, USA) and luminescence reading were measured on a GloMax Luminometer (Promega).

### 2.10 Reverse transcription‐quantitative PCR

Cell lines were harvested, and total RNA was isolated using TRIzol (Invitrogen/Thermo Fisher Scientific) and RNeasy mini kit (QIAGEN) following the manufacturer’s instructions. For RT‐qPCR, cDNA was prepared using the High‐capacity cDNA reverse transcription kit (Applied Biosystems, Waltham, MA, USA). Real‐time quantitative PCR was performed by a LightCycler 480 II (Roche, Indianapolis, IN, USA) using the TaqMan probe primer mix for RUNX1T1 (Thermo Fisher Scientific, catalog # 4331182). Results were normalized to the housekeeping gene beta‐actin.

### 2.11 Chromatin immunoprecipitation

For RUNX1T1 ChIP assays, cross‐linking was performed in two steps: protein–protein complexes were first cross‐linked using disuccinimidyl glutarate (DSG) at 2 mm for 45 min at RT followed by protein–DNA cross‐linking using 1% formaldehyde (Sigma) for 10 min at RT. For pan‐acetylated H3 ChIP assays, protein‐DNA complexes were cross‐linked using 1% formaldehyde for 7 min at RT. Both two‐step and one‐step cross‐linked cells were quenched with glycine at 0.25M for at least 5 min at RT and harvested for chromatin preparation using a Covaris sonicator (Covaris, Woburn, MA, USA). DNA fragment size was checked by agarose gel electrophoresis. Chromatin was precleared using protein A/G magnetic beads (Thermo Fisher Scientific), and 1% of the total chromatin was used as an input for normalization. The chromatin was immunoprecipitated using anti‐FLAG antibody (Sigma, catalog #F1804) or pan‐acetylated H3 antibody (Active Motif, Carlsbad, CA, USA, catalog #39040) at 4°C overnight, and chromatin‐antibody complexes were captured using protein A/G magnetic beads (Thermo Fisher Scientific). Reverse cross‐linking was done overnight at 65°C followed by RNase cocktail (Invitrogen) and protease K treatments. DNA was isolated using the phenol‐chloroform extraction method. The isolated DNA was analyzed by qPCR using the seven primer sets targeting the *CDKN1A* (p21) promoter proximal and distal regions (sequences shown in Table S2).

### 2.12 ChIP‐seq data analysis

ChIP‐seq raw fastq data files were downloaded from Gene Expression Omnibus (GEO) accession number GSE29222 (H3K9ac ChIP‐seq dataset SRR203377 and SRR203378). Reads were aligned against hg38 genome by bowtie2 using sensitive mode parameter (‐D 15 ‐R 2 ‐N 0 ‐L 20 ‐i S,1,0.75). SAM files generated from Bowtie2 were converted into BAM files using SAMtools. BAM files were normalized by depth after removal of PCR duplicates and blacklisted regions. A gene list (downloaded from UCSC table‐browser) was curated after removing noncanonical, mitochondrial, genes shorter than 2 kb and overlapping genes on the same strand, which resulted in 21,396 genes. Heatmaps and metagene plots were generated in ngs.plot.r program using our curated gene list.

### 2.13 *RUNX1T1* in situ hybridization and image analysis

*RUNX1T1* mRNA expression was detected in tumor cores of two different tissue microarray (TMA) slides that contained either c‐SCLC specimens or mixed lung cancer specimens, including ‘pure’ SCLC. *RUNX1T1* in situ hybridization was performed using a chromogenic RNAscope 2.5 HD Duplex Detection Kit (ACDbio, Newark, CA, USA) according to the manufacturer’s instructions. A custom *RUNX1T1* probe that detects all *RUNX1T1* mRNA variants was designed and mixed with a standard *MYC* probe during hybridization to generate red and green signals, respectively.

To quantitate *RUNX1T1* levels in the c‐SCLC tumor, two SCLC cores and two NSCLC cores from the same ‘combined’ SCLC patient tumor were analyzed. Each core was represented by three images randomly selected at 40x magnification. The *RUNX1T1* RNAscope images were evaluated with CellProfiler image analysis software [22]. Specifically, cell segmentation was done using a global thresholding strategy set to a maximum diameter of 100 pixels after deconvolution of the hematoxylin stain, and the *RUNX1T1* signal was isolated by excluding all colors but red (R = 0.05, G = 1, B = 1, background set to black) and detected via global thresholding set to a minimum spot diameter of 3 pixels. Images were scored as dots/cell.

### 2.14 Statistical analysis

For luciferase reporter assays and RT‐qPCR assays, data represent mean ± standard deviation (SD) of the number of experimental replicates indicated in figure legends. Statistical analyses were performed using GraphPad Prism 6. P‐values were calculated by unpaired or multiple t‐tests, as indicated, and *P*‐values ≤ 0.05 were considered significant.

## 3 Results

### 3.1 *RUNX1T1* is specifically amplified in small cell tumors of ‘combined’ SCLC patients

To discover clinically important genetic alterations in SCLC, we routinely perform targeted exome sequencing on our patients with SCLC (currently *N* = 90), which included two patients with c‐SCLC having synchronous SCLC and NSCLC tumors. A close comparison of the specific genetic alterations found in the two patients with c‐SCLC revealed that *RUNX1T1* amplification was detected exclusively in the SCLC tumors (Table 1). By comparison, *RUNX1T1* amplification was rare in the remaining patients with ‘pure’ SCLC, occurring in only 2 of 88 patients. Further, *RUNX1T1* amplification occurred in the absence of *MYC* amplification, which is located on the same chromosomal arm. The threshold for calling amplification is a copy number ≥ 8. In one of the patients with c‐SCLC (PID #512), *RUNX1T1* amplification is the only gene alteration that truly differentiates SCLC from NSCLC tumors. In the other patient (PID #508), there was a more even distribution of shared and histology‐specific mutations between the tumors.

**Table 1:** Genetic alterations in two c‐SCLC cases

Case Biopsy histology (date—site)	PID 508		PID 512	
	SCLC (1/20—diaphragm)	NSCLC (8/4—lymph node)	SCLC (2/26—lung)	NSCLC (2/5—lung)
SCLC‐specific mutations	*CTNNB1*: Q177E, S45F; *PARK2*: P437L; *PREX2*: splice site 3974_3984 + 7delCACCAAACTTGGTAAGGA; **RB1 loss exons 9‐11;** ***RUNX1T1:* amplification;** ***TP53*** : M246I		*ERBB4*: Q206_T207del; *KEAP1*: complex rearrangement; ***RUNX1T1:*** amplification	
Shared mutations	*AURKB*: M58I; *BRIP1*: T1050N; *GPR124*: R448H; *JAK3*: L1073F; ***KRAS*** : G12C		*ALK*: E370K; *AR*: Q799E; *BARD1*: N356I; *BRCA2*: S976I; *CIC*: A756V, G516R; *CTCF*: D194Y; *DAXX*: I127M; *FAM46C*: D182V; *FGF3*: T128M; *FGF10*: amplification; *FGFR1*: amplification; *FH*: P18L; *GPR124*: I41V, amplification; *HGF*: P27Q; *IL7R*: amplification; *IRF4*: A136D; *JAK2*: K244R; *KDM5A*: P328L; *LRP1B*: A3307S; *MAP3K1*: amplification; *MYC*: V282L; *MPL*: T487I; *NOTCH 1*: R731Q; *PRKAR1A*: Y373C; *PRDM1*: E743K; *PTCH1*: T1064M; *SDHA*: amplification; *SMAD2*: amplification; *SPEN*: truncation exon 11; *STK11*: K64*; *RET*: L724F; *RICTOR*: amplification; ***TP53:*** E298*; *ZNF217*: P851L; *ZNF703*: amplification	
NSCLC‐specific mutations		*ALK*: E465*; ***CDKN2A/B:*** loss; *CDK8*: R200*; *FUBP1*: rearrangement exon 16; *MEF2B*: R219Q; *MTOR*: E1507K; *PARK2*: P437L; S*MAD4*: F339L; *STAT3*: E444*; ***TP53:*** S183*		*ERBB4*: splice site 617‐3_622delAGACTTGTA; *KEAP1*: rearrangement exon 3

Bold text highlights mutations of particular note.

### 3.2 *RUNX1T1* expression is upregulated in the SCLC component of a c‐SCLC tumor

**Fig. 1:**
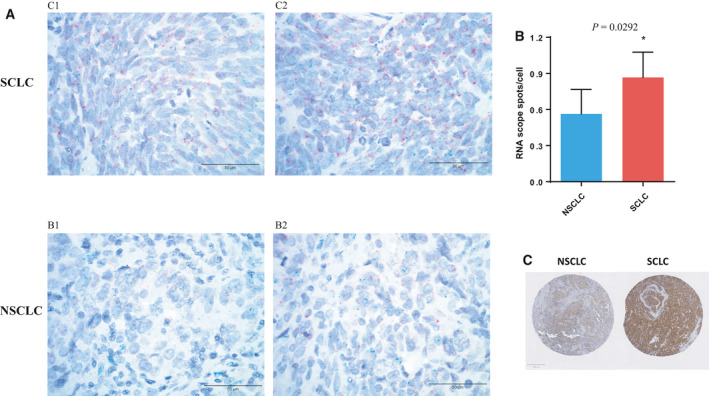
*RUNX1T1* expression is upregulated in the SCLC component of c‐SCLC tumors. (a) Examples of RNAscope in situ hybridization results of cores from a c‐SCLC tumor: two from the SCLC component (upper panel) and two from the NSCLC component (lower panel). Scale bar = 50 µm. *RUNX1T1* mRNA signal is detected as red dots and *MYC* by the green dots. (b) Quantitation of the *RUNX1T1* RNAscope signal of NSCLC and SCLC components (3 images/core for 2 cores of each histology) using CellProfiler. *P = 0.0292 from unpaired t‐test. Error bars represent mean ± SD. (c) INSM1 IHC stain of representative NSCLC and SCLC cores from c‐SCLC patient. Scale bar = 50 µm.

We did not have any additional tumor specimen to validate *RUNX1T1* amplification or expression in our original two c‐SCLC tumors. We did, however, obtain tumor tissue from a third c‐SCLC tumor to validate *RUNX1T1* expression in SCLC. We used chromogenic RNAscope *in situ* hybridization to detect *RUNX1T1* mRNA expression in four tissue cores taken from different regions of the tumor. Each core predominantly displayed either a SCLC or a NSCLC histology. As shown in Fig. 1A, the level of *RUNX1T1* mRNA expression in the SCLC component of the tumor, detected as red dots, is noticeably higher than that observed in the NSCLC component. This difference of *RUNX1T1* expression between histologic components was confirmed by quantitative image analysis (shown in Fig. 1B), where the *RUNX1T1* positive signal was measured as red dots per cell (*P* = 0.0292). The histology differences among the cores were supported by immunohistochemical (IHC) staining for the neuroendocrine marker INSM1, which was much more robust in the SCLC component (see Fig. 1C). Thus, *RUNX1T1* expression was significantly higher in the SCLC component of a third c‐SCLC tumor.

### 3.3 *RUNX1T1* is highly expressed in SCLC

**Fig. 2:**
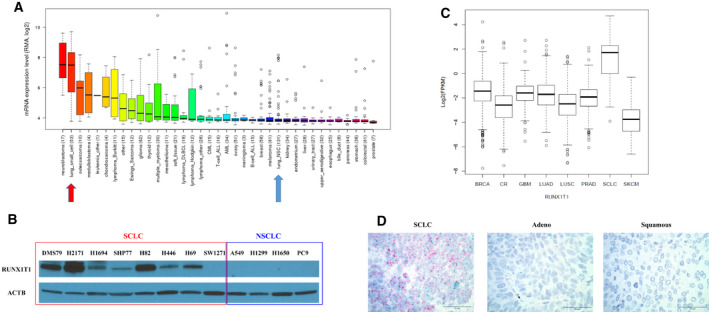
*RUNX1T1* is highly expressed in SCLC. (A) *RUNX1T1* mRNA levels in cancer cell lines based on Affymetrix data from the CCLE. SCLC is highlighted with a red arrow and NSCLC with a blue arrow. (B) Western blot of RUNX1T1 expression in eight SCLC cell lines (DMS79, H2171, H1694, SHP77, H82, H446, H69, and SW1271) and four NSCLC cell lines (A549, H1299, H1650, and PC9); beta‐actin was used as the loading control. (C) *RUNX1T1* mRNA levels in tumor samples detected by RNA‐Seq comparing SCLC with other cancers (BRCA: breast cancer, CR: colorectal cancer, GBM: glioblastoma, LUAD: lung adenoma NSCLC, LUSC: lung squamous NSCLC, PRAD: prostate adenoma cancer, SKCM: skin melanoma cancer). (D) *RUNX1T1* in situ hybridization examples using RNAscope on clinical tumor samples of ‘pure’ SCLC, lung adenocarcinoma and lung squamous cell carcinoma. Red dots identify *RUNX1T1* mRNA signal, green dots *MYC* mRNA signal. Scale bar = 50 µm.

To determine whether *RUNX1T1* expression was higher in general in SCLC compared with NSCLC and other cancers, we initially looked at the expression profile of *RUNX1T1* mRNA using the Broad Cancer Cell Line Encyclopedia (CCLE) database. As shown in Fig. 2A, *RUNX1T1* mRNA is highly expressed among SCLC cell lines compared with most other types of cancers, including non–small‐cell lung cancer (NSCLC). Consistent with *RUNX1T1* mRNA levels, western blot analysis (shown in Fig. 2B) shows high RUNX1T1 protein levels in SCLC cell lines in comparison with NSCLC cell lines.

Additionally, we also compared *RUNX1T1* expression in tumor specimens using RNA‐Seq data from a SCLC genomics study [23] and the TCGA. As shown in Fig. 2C, SCLC shows the highest levels of *RUNX1T1* mRNA among all tumors studied. Lastly, we measured *RUNX1T1* mRNA expression in tissue microarrays (TMA) containing SCLC and NSCLC patient tumor cores by chromogenic RNAscope *in situ* hybridization. Again, a positive *RUNX1T1* mRNA signal was detected as red dots on the tumor sections. SCLC specimens showed a range of *RUNX1T1* positivity, in contrast with lung adenocarcinoma and lung squamous cell tumors, where little to no *RUNX1T1* signal was detected (Fig. 2D). Taken together, these data suggest that *RUNX1T1* expression is specific for SCLC compared with NSCLC.

### 3.4 Overexpression and knockout of *RUNX1T1* leads to significant changes in gene expression in hallmark pathways

**Fig. 3:**
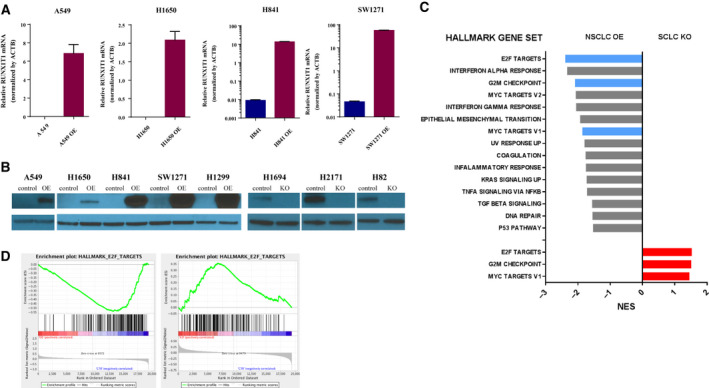
Overexpression and knockout of *RUNX1T1* lead to significant changes in hallmark pathways. (A) *RUNX1T1* RT‐qPCR validating overexpression efficiency in cell lines (*n* = 3 replicates of individual experiment/cell, error bars represent mean ± SEM). The house keep gene beta‐actin was used for normalization. No signal was detected in parental A549 and H1650 cells. (B) Western blot results validating RUNX1T1 overexpression (OE) and knockout (KO) in various cell lines. (C) Significantly changed hallmark pathways identified by GSEA after *RUNX1T1* overexpression in NSCLC cell lines (H1299 and H1650) or *RUNX1T1* knockout in SCLC cell lines (H82 and H2171). Blue and red bars show the overlapped pathways inversely affected by overexpression and knockout, respectively. NES: normalized enrichment score. (D) GSEA plots showing ‘E2F TARGETS’ genes depleted by *RUNX1T1* overexpression in NSCLC (H1650 and H1299) and enriched by *RUNX1T1* knockout in SCLC (H82 and H2171).

To explore potential downstream function(s) of RUNX1T1, we used lentivirus to stably overexpress *RUNX1T1* in five lung cancer cell lines with low endogenous *RUNX1T1* expression (A549, H16450, H841, SW1271, H1299) to create models of *RUNX1T1* amplification. To complement these overexpression models, we also stably knocked out *RUNX1T1* in three SCLC cell lines with high endogenous *RUNX1T1* mRNA expression levels (H1694, H2171, H82) using CRISPR/Cas9. Overexpression and knockout of *RUNX1T1* in these cell line models were validated by RT‐qPCR (shown in Fig. 3A) and western blotting (shown in Fig. 3B).

Overexpression or knockout of *RUNX1T1* in our cell line models did not produce obvious histologic changes or consistent alterations in the expression of several neuroendocrine (NE) markers (ASCL1, NeuroD1, synaptophysin, INSM1, and TTF1) assayed by western blot analysis, as might be expected since NE gene expression is a feature distinguishing SCLC from NSCLC (data not shown). Therefore, we performed microarray analysis to look for changes in the mRNA expression profiles of three of the *RUNX1T1* overexpressing cell lines (H1650, SW1271, H1299) and two of the *RUNX1T1* knockout cell lines (H2171, H82) compared with their corresponding controls. Hierarchal clustering analyses did not show any dramatic shifts toward a common expression profile in either the *RUNX1T1* overexpressing or knockout cell lines (Fig. S1). However, gene set enrichment analysis (GSEA) of hallmark genes identified multiple pathways that were significantly (*P* < 0.05) downregulated by *RUNX1T1* overexpression in NSCLC cell lines (H1650, H1299) or upregulated by *RUNX1T1* knockout in SCLC cell lines (H82, H2171) (shown in Fig. 3C, see Figures S2 and S3 for details). We focused on these directional changes because of the putative role of RUNX1T1 as a transcriptional co‐repressor. Among these pathways, we found that the ‘E2F TARGETS’ pathway ranked as the most significantly changed pathway that was inversely regulated in *RUNX1T1* overexpressing versus *RUNX1T1* knockout cell lines (shown Fig. 3D). All future experiments focused on the effects of *RUNX1T1* overexpression because this model was most relevant to our original observation of *RUNX1T1* amplification in c‐SCLC.

### 3.5 *RUNX1T1* overexpression increases E2F activity and decreases *CDKN1A* (p21) expression

**Fig. 4:**
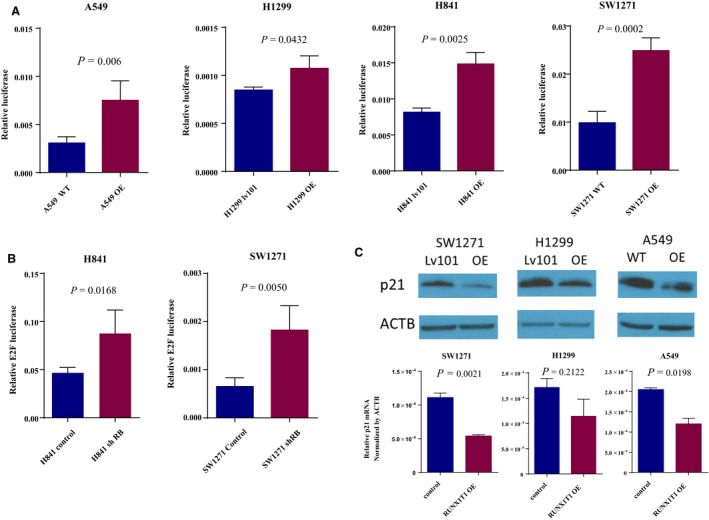
*RUNX1T1* overexpression increases E2F activity and decreases *CDKN1A* (p21) expression. (A) E2F luciferase reporter assays in *RUNX1T1* overexpressing cell lines A549, H1299, H841, and SW1271 (*n* = 4 replicates of individual experiment/cell, 2 experiments/cell). Error bars represent mean ± SD. Significance by unpaired multiple t‐test. (b) E2F luciferase activity in H841 and SW1271 cells with stable knockdown of *RB1* by shRNA (*n* = 4 replicates of individual experiment/cell). Error bars represent mean ± SD. Significance by unpaired t‐test (c) *CDKN1A* (p21) western blot and RT‐qPCR (*n* = 3 replicates of individual experiment/cell, error bars represent mean ± SEM) in *RUNX1T1* overexpressing (OE) cells compared with the corresponding control. Beta‐actin was used as the loading control in western blotting and for normalization in qPCR. Significance by unpaired *t*‐test.

To validate our bioinformatics results, we used luciferase reporter assays to detect changes in E2F pathway activity in *RUNX1T1* overexpressing cells. As shown in Fig. 4A, the overexpression of *RUNX1T1* paradoxically increased E2F reporter activity in four different cell lines, suggesting that RUNX1T1 promotes E2F transcriptional activity. As a control, we could show that E2F pathway activity is similarly increased in two of the same cell lines (H841 and SW1271) when *RB1* is stably knocked down with shRNA (shown in Fig. 4B). This indicates that overexpression of *RUNX1T1* produces a phenotype similar to *RB1* loss, which is a common genomic alteration in SCLC.

To discover a potential mechanism behind the increased E2F activity induced by *RUNX1T1* overexpression, we re‐examined the microarray data and found that *CDKN1A* (p21) expression was the most downregulated E2F target gene after *RUNX1T1* overexpression (Fig. S4a). Interestingly, *CDKN1A* (p21) has been reported as both a downstream target of E2F activity and an upstream negative regulator of E2F signaling, making it a model gene to delineate a mechanism of action for overexpressed RUNX1T1 [24]. Thus, we used western blotting and RT‐qPCR to initially validate that *RUNX1T1* overexpression decreases *CDKN1A* (p21) expression (Fig. 4C).

### 3.6 RUNX1T1 binding to the *CDKN1A* (p21) promoter parallels altered histone acetylation

**Fig. 5:**
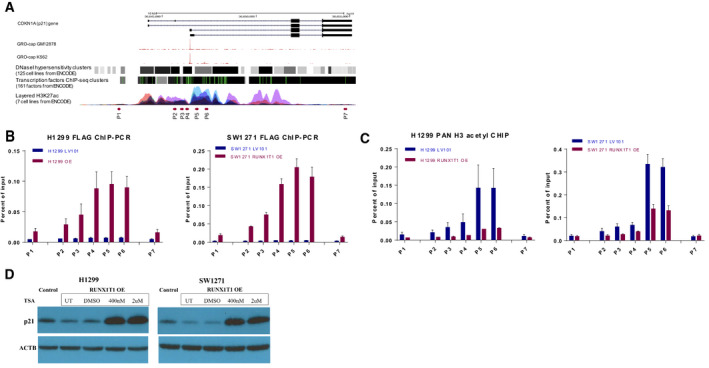
*RUNX1T1* overexpression decreases histone acetylation at the *CDKN1A* (p21) promoter. (A) UCSC browser tracks showing *CDKN1A* gene annotation and precise transcription start site (TSS), as shown in GRO‐cap tracks of two different cell lines. DNaseI hyposensitivity and transcription factor ChIP‐seq tracks from ENCODE indicate tight regulation of the p21 gene. Enrichment of H3K27 ac (ENCODE ChIP‐seq data) and location of primer sets used in ChIP‐qPCR analysis are indicated. (B) RUNX1T1 ChIP‐qPCR results in overexpressing (OE) vs control H1299 and SW1271 cells targeting seven *CDKN1A* (p21) promoter and distal regions (*n* = 3 replicates of biological duplicates/cell). Error bars represent mean ± SD (C) ChIP‐qPCR of pan‐histone 3 acetylation in OE vs control H1299 and SW1271 cells targeting the same seven *CDKN1A* (p21) promoter and distal regions (*n* = 3 replicates of biological duplicates/cell). Error bars represent mean ± SD. (D) Western blot of *CDKN1A* (p21) in H1299 and SW1271 under Trichostatin A (TSA) treatment at 400nM and 2µM for six hours. Beta‐actin was used as the loading control. UT: untreated. DMSO: DMSO vehicle control.

To determine whether RUNX1T1 can bind to the *CDKN1A* (p21) gene promoter and regulate its transcription, we performed chromatin immunoprecipitation (ChIP) assays using an anti‐FLAG antibody to pull down RUNX1T1‐chromatin complexes in H1299 and SW1271 cell lines. ChIP DNA fragments were analyzed by qPCR using seven sets of primers covering regions both proximal and distal to the transcription start site (TSS) of *CDKN1A* (p21) that are shown in the ENCODE database (https://www.encodeproject.org/) to be active sites for transcription factor binding and epigenetic regulation (Fig. 5A). As shown in Fig. 5B, the detection of target DNA fragments proximal to the TSS of the *CDKN1A* (p21) promoter (P4, P5, P6) was dramatically increased in RUNX1T1 overexpressing cells relative to controls, suggesting that RUNX1T1 extensively binds to the *CDKN1A* (p21) promoter at these regions. As an internal negative control, RUNX1T1 ChIP signals to the distal regions of *CDKN1A* (p21) promoter (P1, P7) were not enriched in overexpressing cells relative to control cells. Because RUNX1T1 is known to interact with histone deacetylases (HDACs) on its own [7, 9], we performed parallel ChIP experiments using a pan‐acetylated histone H3 antibody in the same cell lines (H1299 and SW1271). We found that regions with high RUNX1T1 binding showed dramatic decreases in histone H3 acetylation (shown in Fig. 5C).

Our data suggest that RUNX1T1 binding is associated with decreased histone acetylation at target gene promoters and may be responsible for the decrease in *CDKN1A* (p21) expression observed in RUNX1T1‐overexpressing cells. To investigate this idea, we treated RUNX1T1 overexpressing cells with the HDAC inhibitor Trichostatin A (TSA) and could show that *CDKN1A* (p21) expression was dramatically restored in RUNX1T1‐overexpressing H1299 and SW1271 cell lines (shown in Fig. 5D).

**Fig. 6:**
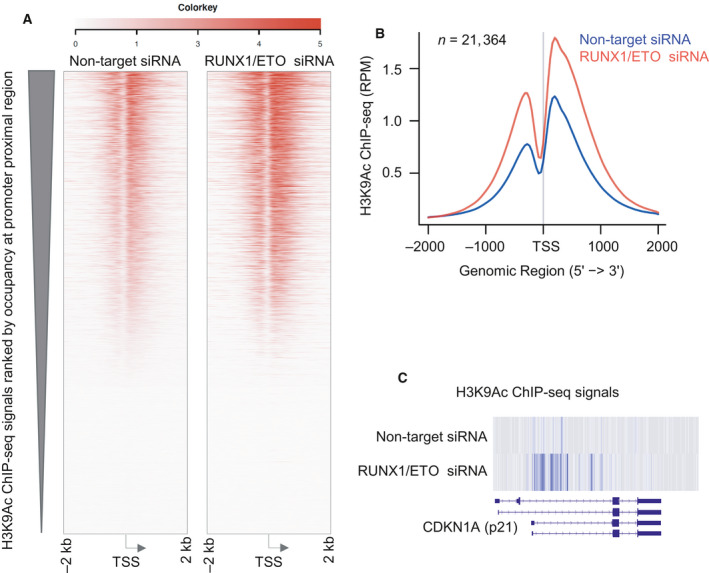
*RUNX1‐RUNX1T1* (AML/ETO) knockdown increases global H3K9 acetylation. Heatmap (A) and metagene plot (B) showing the enrichment of H3K9ac near the TSS of 21,364 curated genes in Kasumi‐1 cells (H3K9ac ChIP‐seq dataset SRR203377 and SRR203378). (C) H3K9ac level at *CDKN1A* promoter proximal region is shown using IGV (integrated genome viewer).

RUNX1T1 function has been mostly studied in the context of its fusion protein, AML1‐ETO. To determine whether RUNX1T1 binding in this context is associated with changes in histone acetylation, we reanalyzed ChIP‐seq data from GEO accession number GSE29222, in which the effect of siRNA knockdown of the *RUNX1‐RUNX1T1* fusion transcript on global histone acetylation was examined in Kasumi‐1 AML cells. Indeed, our reanalysis revealed that siRNA treatment increased global H3K9 acetylation (Fig. 6A), and this effect was largely centered on transcription start sites (TSS) (Fig. 6B). Importantly, the *CDKN1A* (p21) promoter specifically demonstrated increased H3K9 acetylation after siRNA treatment (Fig. 6C).

## 4 Discussion

Recent studies have made a lot of progress in understanding the molecular mechanism(s) of SCLC development and progression [1, 3]. Dual inactivation of the *TP53* and *RB1* genes has been found in > 80% of all ‘pure’ SCLC tumors [2, 25] and has been proven to play a key role in genetically engineered mouse models (GEMMs) of this disease. In addition, other genes such as *RBL2, PTEN,* and *MYC* have been found to play contributing roles in SCLC tumorigenesis [26, 27, 28]. These studies, however, have all focused on so‐called ‘pure’ SCLC. Here, we initially focused on the other clinically important subgroup of SCLC, called ‘combined’ SCLC, about which little is known. The reason to study c‐SCLC, however, is that its diagnosis changes the clinical treatment of lung cancer from a NSCLC to a SCLC approach. Thus, new therapeutic targets for all SCLC may be identified by understanding the unique molecular pathology of c‐SCLC.

We identify *RUNX1T1* as an amplified gene in SCLC, but not in NSCLC, tumors of two c‐SCLC cases in our cohort. Further RNAscope analysis of an additional c‐SCLC tumor specimen also found significantly higher *RUNX1T1* mRNA expression in the SCLC component compared with the NSCLC component. These results are consistent with the higher *RUNX1T1* mRNA and protein expression observed in ‘pure’ SCLC cell lines and tumors compared with NSCLC. Thus, our findings indicate for the first time that *RUNX1T1* expression may be a biomarker for the SCLC phenotype. This idea is supported by the co‐expression of *RUNX1T1* with classic neuroendocrine (NE) markers (*ASCL1*, *INSM1*, *NEUROD1, SYP*) in SCLC cell lines annotated in the CCLE database (Fig. S5). This does not exclude, however, a potential role for *RUNX1T1* in SCLC tumorigenesis as ASCL1 is a transcription factor that is both a classic NE biomarker for SCLC and has been shown in GEMMs to be essential for SCLC development [29].

Clearly, the underlying mechanism(s) driving high *RUNX1T1* expression in ‘pure’ versus c‐SCLC, that is, with or without gene amplification, appears different. Because of the paucity of c‐SCLC cases, however, it is impossible to draw firm conclusions in this context. The only two previous genomic sequencing studies of c‐SCLC, which were also limited to cohorts of four [30] and three [6] patients, are not helpful in this regard because they used targeted exome sequencing platforms that did not include *RUNX1T1*.

Regardless of the mechanism, we wondered whether high *RUNX1T1* expression could play any role in shaping the SCLC phenotype. Our GSEA analysis of microarray data in two NSCLC cell lines (H1650 and H1299) identified significant downregulation of the E2F TARGETS hallmark gene set after overexpressing RUNX1T1 (Fig. 3C,D). Interestingly, after validation of seven E2F target genes in more RUNX1T1 overexpressing cell lines (H1650, H1299, SW1271 and A549) using RT‐qPCR, we found that the best downregulation of E2F target genes is observed in H1650 cells and was less consistent in the other three cell lines (Fig. S4b). This suggested that the H1650 results might be driving the downregulation of E2F TARGETS detected by GSEA hallmark analysis of the microarray results performed in only H1650 and H1299 cell lines. Thus, we used E2F luciferase reporter assays as a different approach to investigate, and potentially validate, alteration of E2F pathway activity by RUNX1T1. This assay can integrate the regulatory signals of many E2F family members and therefore represents a more global reporter of E2F transcriptional activity. This led us to discover a consistent increase in E2F transcriptional activity associated with *RUNX1T1* overexpression in multiple cell lines (H1299, A549, SW1271, and H841). Interestingly, increased E2F activity is an expected feature of SCLC due to the recurrent loss of the negative regulator *RB1* in this cancer [3]; and we were able to confirm this phenotype in our own *RB1* knockdown models (shown in Fig. 4B). The increase in E2F transcriptional activity generated by *RUNX1T1* overexpression in *RB1* sufficient cells is intriguing and suggests one mechanism whereby *RUNX1T1* may contribute to a SCLC phenotype at a molecular level. In this regard, it is interesting to note that *RB1* loss was not detected in one of our three c‐SCLC cases (Table 1 and Table S1), suggesting that it may not be required in c‐SCLC formation. Furthermore, there were no detectable genomic *RB1* alterations in 2 of 4 [30] and 3 of 3 [6] cases of c‐SCLC in two previous studies. Parallel immunohistochemical (IHC) analyses in one of these studies also demonstrated positive RB1 staining in one of the two wild‐type *RB1* c‐SCLC cases [30]. Thus, the role of *RB1* loss in c‐SCLC remains unclear.

Another attraction to study *RUNX1T1* in SCLC was because of its reported role as an epigenetic regulator [7, 9, 10, 11]. Furthermore, an epigenetic role for RUNX1T1 by itself as a nonfusion protein has rarely been examined in cancer; thus, any finding in lung carcinoma would be novel. Indeed, our results support this idea as RUNX1T1 binding was associated with decreased histone acetylation at the *CDKN1A* (p21) promoter in RUNX1T1‐overexpressing cells (Fig. 5B). Additionally, *CDKN1A* (p21) expression is dramatically restored by HDAC inhibition in RUNX1T1‐overexpressing cells (Fig. 5D). These results are consistent with previous studies showing that *CDKN1A* (p21) gene expression is actively regulated through histone acetylation/deacetylation at its promoter region [31, 32]. Taken together, our data support a model where RUNX1T1 assembles in HDAC repressor complexes that bind to target gene promoter regions and inhibit transcription via decreased histone acetylation.

In the context of its fusion protein, AML1‐ETO, AML1 provides the DNA‐binding domain, and ETO (RUNX1T1) is thought to anchor the assembly of transcriptional repressor complexes. Although we show binding of RUNX1T1 to the *CDKN1A* (p21) gene (Fig. 5B), previous studies demonstrated no evidence of DNA‐binding ability mediated by RUNX1T1 [7, 11], indicating a need for other transcription factors to localize RUNX1T1 to the *CDKN1A* (p21) promoter. The ENCODE database lists an extensive number of transcription factors that bind to the *CDKN1A* (p21) promoter region (see Fig. 5A). Studies to identify the DNA‐binding partner directing RUNX1T1 to specific gene targets are ongoing; however, we did not find any evidence to support a role for AML1 itself in co‐immunoprecipitation experiments (data not shown).

Our study has identified *RUNX1T1* as a biomarker of SCLC that may be recurrently amplified in c‐SCLC. We show that RUNX1T1 is a potential epigenetic regulator of *CDKN1A* (p21) signaling, and this may serve as a model for how RUNX1T1 regulates the expression of other target genes in SCLC. Although perturbations in *RUNX1T1* expression produced no obvious phenotypic changes in our study, recent discoveries in GEMMs show that multiple epigenetic modifiers (*NFIB*, *EZH2*, and *CREBBP/EP300*) are involved in SCLC development and progression [33, 34, 35, 36]. Indeed, studies of c‐SCLC tumors, although limited by sample size, including our own, have shown more similarities than differences in the genomic mutation profiles of the different histologic components [6, 30], suggesting that epigenetic regulation could play a larger role in lung cancer phenotypes.

## 5 Conclusion

Our study has identified *RUNX1T1* amplification in SCLC component of patients with c‐SCLC, as well as high *RUNX1T1* expression in SCLC. Further investigation reveals that *RUNX1T1* overexpression consistently downregulates *CDKN1A* (p21) expre

ssion and promotes E2F transcriptional activity in lung cancer cell lines. We have found that overexpressed RUNX1T1 interacts with *CDKN1A* (p21) promoter and is associated with dramatically reduced histone H3 acetylation. Moreover, downregulated *CDKN1A* (p21) by *RUNX1T1* overexpression can be restored by HDAC inhibition. Taken together, our findings suggest that RUNX1T1 is a potential marker and an epigenetic modifier in SCLC.

## **Author** **contributions**

TH, GW, AA, and AD conceived and designed the project and wrote the manuscript. TH and AA performed ChIP experiments and analyses. AK and MY assembled and analyzed TMAs by RNA scope. TH, AS, KM, and CM performed and analyzed experiments, and created stable cell lines. ACT performed bioinformatics analysis of gene arrays. KS performed image analyses on RNA scope experiments. TH, GW, AK, ACT, KS, and AA produced graphical figures. AD provided general oversight of project.

## Conflict of Interest

The authors declare no potential conflicts of interest.

### Peer Review

The peer review history for this article is available at https://publons.com/publon/10.1002/1878‐0261.12829.

## Supporting information


**Fig S1.** Hierarchal clustering analyses based on the microarray data after *RUNX1T1* knockout in cell lines H2171 and H82, and *RUNX1T1* overexpression in cell lines H1650, SW1271, and H1299.

**Fig S2.** Hallmark gene sets depleted in *RUNX1T1*‐overexpressing cells. GSEA pathway analysis identified downregulated hallmark gene sets after *RUNX1T1* overexpression in cell lines H1650 and H1299.

**Fig S3.** Hallmark gene sets enriched in *RUNX1T1* knockout cells. GSEA pathway analysis identified upregulated hallmark gene sets after *RUNX1T1* knockout in cell lines H2171 and H82.

**Fig S4.** E2F target genes affected by *RUNX1T1* overexpression.

**Fig S5.** Correlated expression of *RUNX1T1* with four classic neuroendocrine markers, respectively (*ASCL1*, *NEUROD1*, *INSM1*, *SYP*), in SCLC cell lines annotated in the CCLE database.

**Table S1.** Demographic features of SCLC cohort (N=90).
**Table S2.** qPCR Primer sets for ChIP analyses.
**Table S3.** Gene alterations in the third c‐SCLC patient.
**Table S4.** Collapsed gene list of microarray analysis.
