# Supplementary figures and images for: Identification of RUNX1T1 as a potential epigenetic modifier in small‐cell lung cancer
Source: Mol Oncol. 2020 Nov 27;15(1):195–209. doi: 10.1002/1878-0261.12829 (PMC7782087; doi:10.1002/1878-0261.12829)

Supplement Figure 1

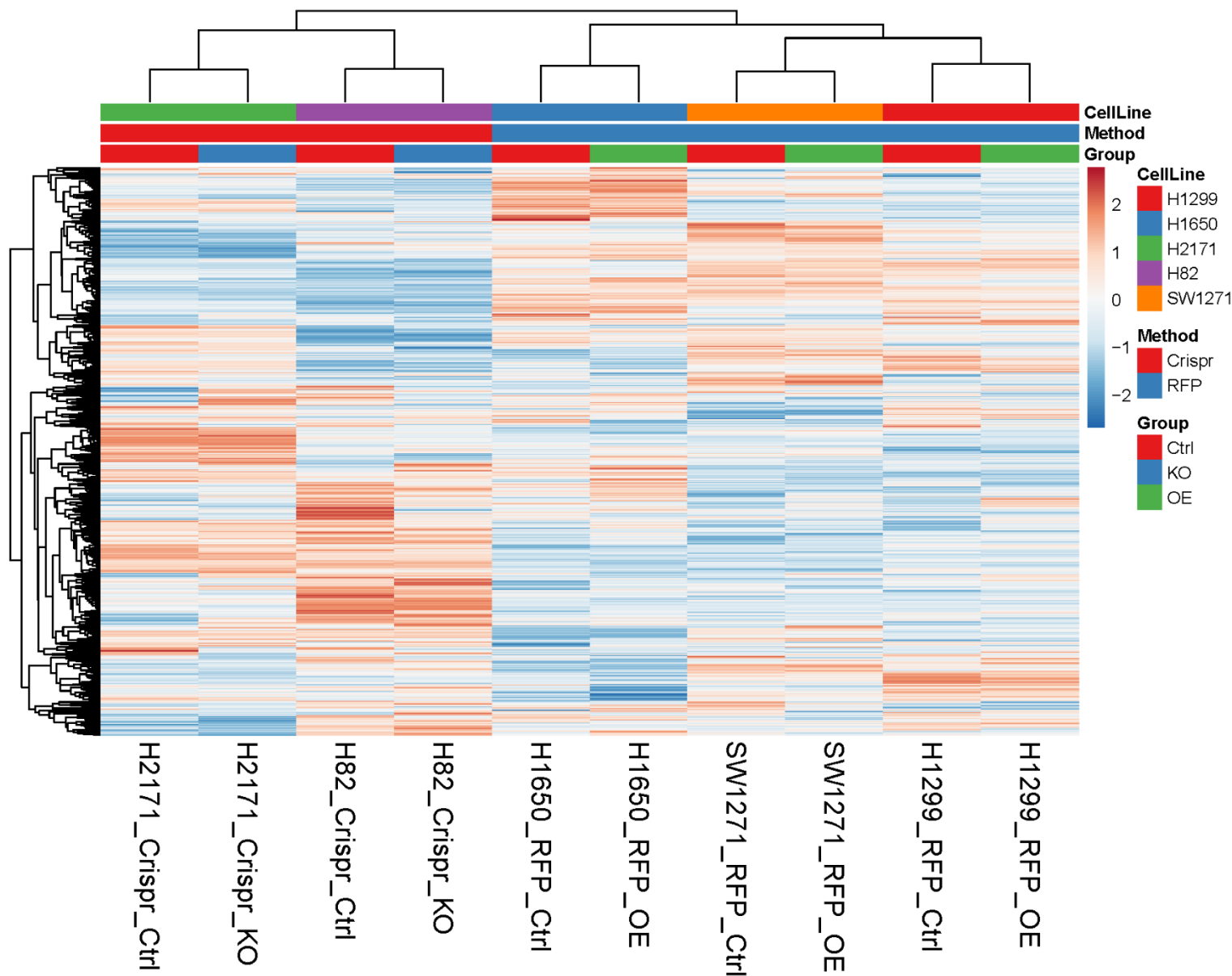

Supplement: Supplementary file 1 — Fig S1. Hierarchal clustering analyses based on the microarray data after RUNX1T1 knockout in cell lines H2171 and H82, and RUNX1T1 overexpression in cell lines H1650, SW1271, and H1299. [file MOL2-15-195-s001.pdf]

# Supplement Figure 4

a

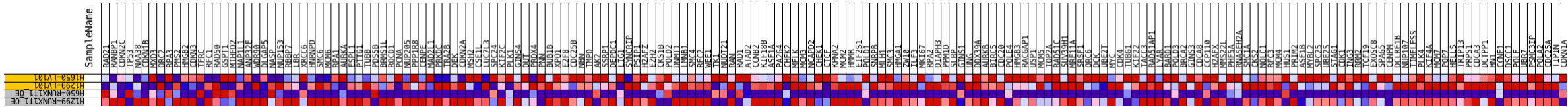

b

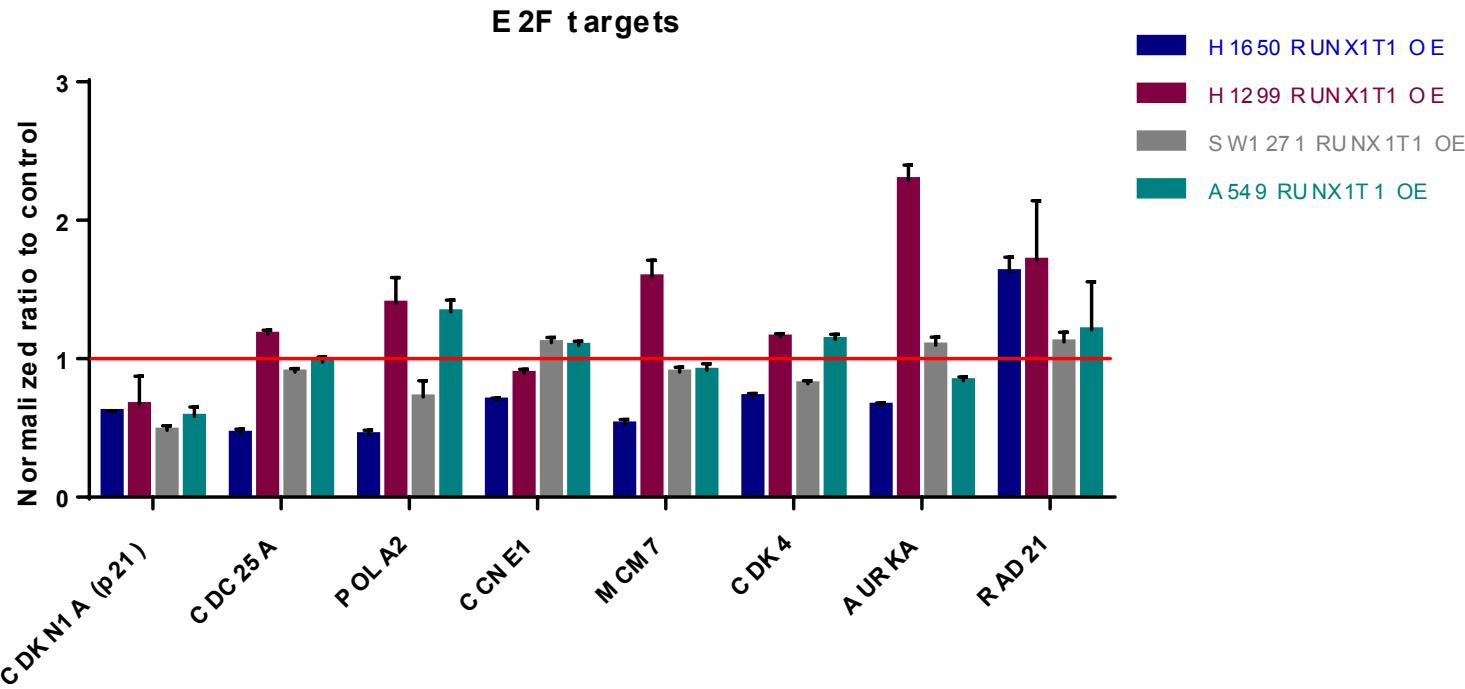

Supplement: Supplementary file 4 — Fig S4. E2F target genes affected by RUNX1T1 overexpression. [file MOL2-15-195-s004.pdf]

Supplement Figure 5

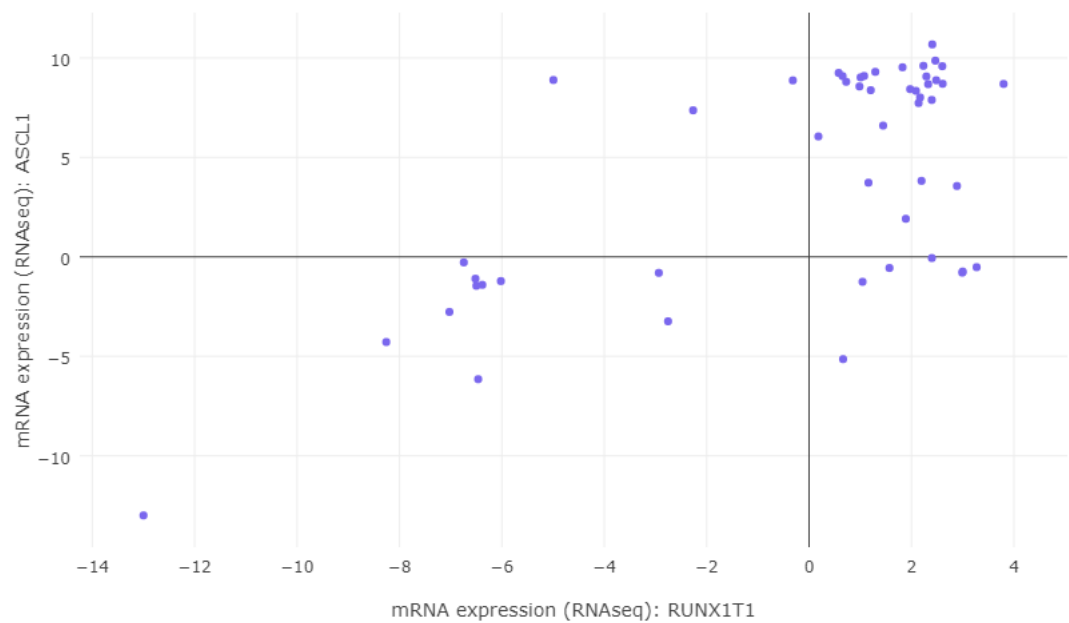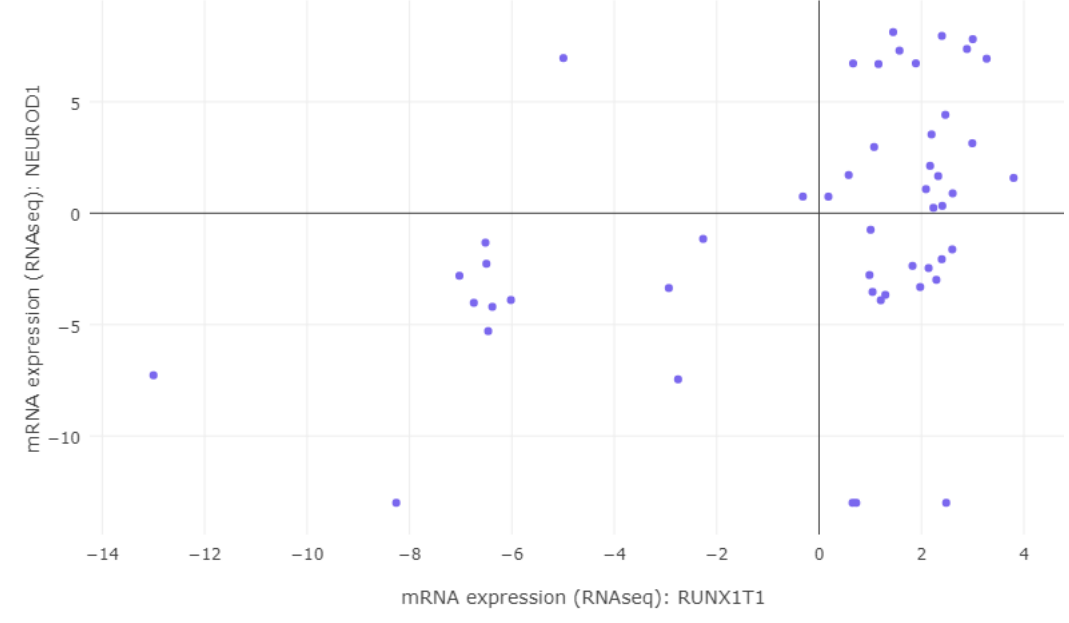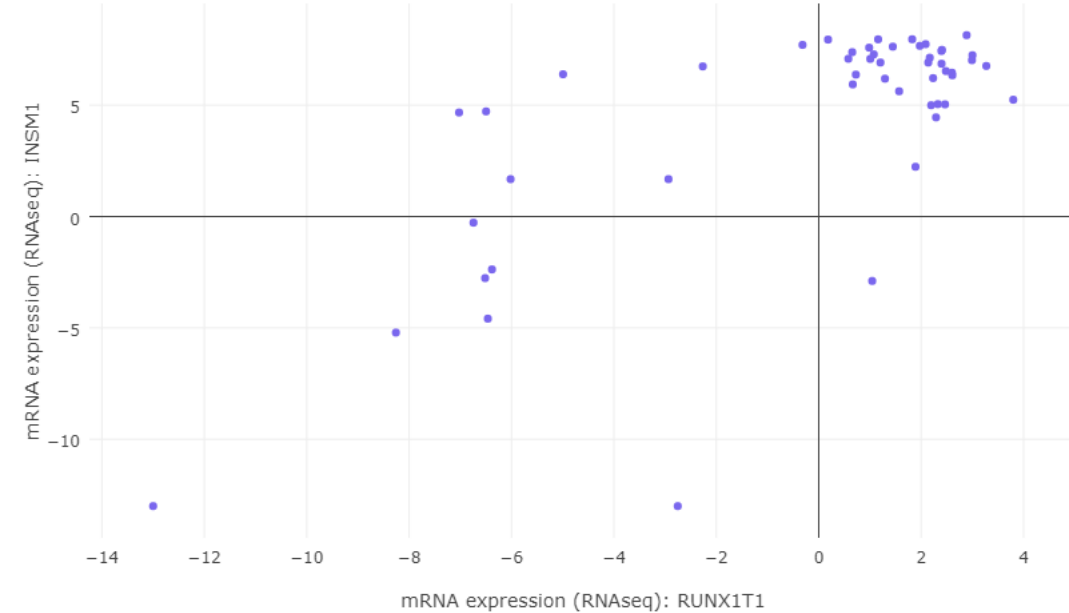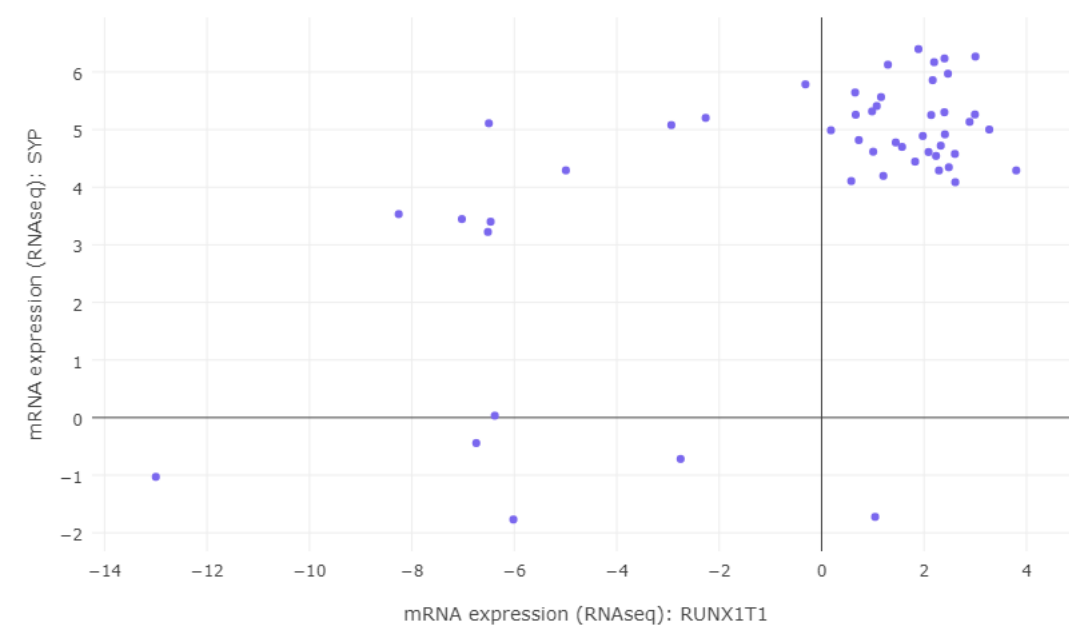

Supplement: Supplementary file 5 — Fig S5. Correlated expression of RUNX1T1 with four classic neuroendocrine markers, respectively (ASCL1, NEUROD1, INSM1, SYP), in SCLC cell lines annotated in the CCLE database. [file MOL2-15-195-s005.pdf]
