# Supplementary material for: Identification of RUNX1T1 as a potential epigenetic modifier in small‐cell lung cancer
Source: Mol Oncol. 2020 Nov 27;15(1):195–209. doi: 10.1002/1878-0261.12829 (PMC7782087; doi:10.1002/1878-0261.12829)
Supplement: Supplementary file 2 — Fig S2. Hallmark gene sets depleted in RUNX1T1‐overexpressing cells. GSEA pathway analysis identified downregulated hallmark gene sets after RUNX1T1 overexpression in cell lines H1650 and H1299. [file MOL2-15-195-s002.pdf]

Supplement Figure 2

NAME	SIZE	NES	NOM p-val
HALLMARK_E2F_TARGETS	185	-2.3909	0.0000
HALLMARK_INTERFERON_ALPHA_RESPONSE	91	-2.3311	0.0000
HALLMARK_G2M_CHECKPOINT	188	-2.0883	0.0000
HALLMARK_MYC_TARGETS_V2	51	-2.0649	0.0000
HALLMARK_INTERFERON_GAMMA_RESPONSE	192	-2.0530	0.0000
HALLMARK_EPITHELIAL_MESENCHYMAL_TRANSITION	191	-1.9321	0.0000
HALLMARK_MYC_TARGETS_V1	178	-1.8589	0.0000
HALLMARK_UV_RESPONSE_UP	154	-1.7959	0.0000
HALLMARK_COAGULATION	132	-1.7601	0.0000
HALLMARK_INFLAMMATORY_RESPONSE	193	-1.7519	0.0000
HALLMARK_KRAS_SIGNALING_UP	190	-1.7283	0.0000
HALLMARK_TNFA_SIGNALING_VIA_NFKB	193	-1.7143	0.0000
HALLMARK_TGF_BETA_SIGNALING	53	-1.5609	0.0101
HALLMARK_DNA_REPAIR	135	-1.5505	0.0020
HALLMARK_P53_PATHWAY	190	-1.5305	0.0039
HALLMARK_ESTROGEN_RESPONSE_LATE	193	-1.4659	0.0020
HALLMARK_XENOBIOTIC_METABOLISM	192	-1.3595	0.0192
HALLMARK_APOPTOSIS	154	-1.3487	0.0100
HALLMARK_ESTROGEN_RESPONSE_EARLY	191	-1.3050	0.0277
HALLMARK_UV_RESPONSE_DN	136	-1.2986	0.0467
HALLMARK_COMPLEMENT	187	-1.2691	0.0444
HALLMARK_IL2_STAT5_SIGNALING	192	-1.1512	0.1429
HALLMARK_APICAL_JUNCTION	192	-1.1345	0.1759
HALLMARK_IL6_JAK_STAT3_SIGNALING	85	-1.1108	0.2570
HALLMARK_SPERMATOGENESIS	130	-1.0494	0.3360
HALLMARK_ANGIOGENESIS	34	-1.0071	0.4380
HALLMARK_MITOTIC_SPINDLE	194	-0.9190	0.7082
HALLMARK_ANDROGEN_RESPONSE	95	-0.8958	0.6546
HALLMARK_NOTCH_SIGNALING	31	-0.7899	0.7896
HALLMARK_UNFOLDED_PROTEIN_RESPONSE	100	-0.7687	0.9259
HALLMARK_WNT_BETA_CATENIN_SIGNALING	41	-0.7585	0.8565
HALLMARK_APICAL_SURFACE	43	-0.7250	0.8922
