# Supplementary material for: Identification of RUNX1T1 as a potential epigenetic modifier in small‐cell lung cancer
Source: Mol Oncol. 2020 Nov 27;15(1):195–209. doi: 10.1002/1878-0261.12829 (PMC7782087; doi:10.1002/1878-0261.12829)
Supplement: Supplementary file 3 — Fig S3. Hallmark gene sets enriched in RUNX1T1 knockout cells. GSEA pathway analysis identified upregulated hallmark gene sets after RUNX1T1 knockout in cell lines H2171 and H82. [file MOL2-15-195-s003.pdf]

Supplement Figure 3

NAME	SIZE	NES	NOM p-val
HALLMARK_E2F_TARGETS	185	1.5276	0.0000
HALLMARK_G2M_CHECKPOINT	188	1.5059	0.0000
HALLMARK_MYC_TARGETS_V1	178	1.4599	0.0000
HALLMARK_COMPLEMENT	187	1.0959	0.2271
HALLMARK_MTORC1_SIGNALING	194	0.9868	0.4829
HALLMARK_PANCREAS_BETA_CELLS	39	0.9416	0.5585
HALLMARK_MYC_TARGETS_V2	51	0.9305	0.5845
HALLMARK_APICAL_JUNCTION	192	0.9140	0.7036
HALLMARK_UV_RESPONSE_UP	154	0.8710	0.8333
HALLMARK_ANGIOGENESIS	34	0.8394	0.7256
HALLMARK_HEDGEHOG_SIGNALING	33	0.6579	0.9777
HALLMARK_OXIDATIVE_PHOSPHORYLATIO	191	0.6260	1.0000
HALLMARK_UNFOLDED_PROTEIN_RESPON	100	0.4133	1.0000
