# Supplementary material for: Identification of RUNX1T1 as a potential epigenetic modifier in small‐cell lung cancer
Source: Mol Oncol. 2020 Nov 27;15(1):195–209. doi: 10.1002/1878-0261.12829 (PMC7782087; doi:10.1002/1878-0261.12829)
Supplement: Supplementary file 6 — Table S1. Demographic features of SCLC cohort (N=90). Table S2. qPCR Primer sets for ChIP analyses. Table S3. Gene alterations in the third c‐SCLC patient. Table S4. Collapsed gene list of microarray analysis. [file MOL2-15-195-s006.docx]

**Supplementary Table 1:** Demographic features of SCLC cohort (N=90)

| **Age at Diagnosis (y)**  (average + SD) | 64.9 + 10.1 |
| --- | --- |
| **Sex**  Male  Female | 43  47 |
| **Race**  White  African-American  Asian  N/A | 54  19  1  16 |
| **Smoking history**  Current  Former  Never  N/A | 39  42  7  2 |
| **VA subtype**  Extensive  Limited  N/A | 70  19  1 |

N/A: not available

**Supplementary Table 2:** qPCR Primer sets for ChIP analyses

| Primer Set | Sequence |
| --- | --- |
| P5 | Forward: TGC GTT CAC AGG TGT TTC T  Reverse: CTG TAC TTG TAA TCC CGC TCT C |
| P6 | Forward: GGA AAG CGG AGT GGA GTA AG  Reverse: TGG ACA CAG TGG CGT AAA G |
| P4 | Forward: AGA GTG CCA ACT CAT TCT CC  Reverse: GTG CGC TGG ACA CAT TTC |
| P3 | Forward: TGA GGG ATC AGT GGG AAT AGA  Reverse: CTC TGG CAG GCA AGG ATT TA |
| P2 | Forward: GAG TGT AGG GTG TAG GGA GAT T  Reverse: AGG AGG GAA TTG GAG AGA CTA C |
| P1 | Forward: GAGGATGCCTCTCTTCAAACA  Reverse: CATGCTCCCGCAGATCTATATT |
| P7 | Forward: CGGAACAAGGAGTCAGACATT  Reverse: AGTGCCAGGAAAGACAACTAC |

**Supplementary Table 3:** Gene alterations in the 3rd c-SCLC patient.

| Gene symbol | Alterations |
| --- | --- |
| *RB1* | Splice site 1154_1215+33del95 |
| *TP53* | R342* |
| *JAK1* | M828I |
| *CIC* | A536V |
| *CREBBP* | Q2216_G2217insQ |
| *ERBB2* | P523S |
| *FAM46C* | C88Y |
| *FANCA* | G811A |
| *FGF14* | G42C |
| *NT5C2* | H486D |
| *PIK3CB* | I458L |
| *RAD51C* | P21A |
| *ZNF703* | G527C |
